# Employment stability and mental health in Spain: towards understanding the influence of gender and partner/marital status

**DOI:** 10.1186/s12889-018-5282-3

**Published:** 2018-04-02

**Authors:** Imma Cortès-Franch, Vicenta Escribà-Agüir, Joan Benach, Lucía Artazcoz

**Affiliations:** 1 Agència de Salut Pública de Barcelona, Pl Lesseps 1, ES-08023 Barcelona, Spain; 2 CIBER en Epidemiología y Salud Pública (CIBERESP), Barcelona, Spain; 3 Institute of Biomedical Research (IIB-Sant Pau), Barcelona, Spain; 4 Programa de doctorat en Metodologia de la Recerca Biomèdica i Salut Pública, Universitat Autònoma de Barcelona, Barcelona, Spain; 5 Universitat Pompeu Fabra, Barcelona, Spain; 6 Centre for Public Health Research (Health Inequalities Area), Valencia, Spain; 7 Nursing Department, Valencian School for Health Studies. Regional Ministry of Health. Generalitat Valenciana, University of Valencia, Valencia, Spain; 8 Health Inequalities Research Group, Employment Conditions Knowledge Network (GREDS-EMCONET), Department of Political and Social Sciences, Universitat Pompeu Fabra, Barcelona, Spain; 9 Johns Hopkins University - Pompeu Fabra University Public Policy Center, Barcelona, Spain; 10 Transdisciplinary Research Group on Socioecological Transitions (GinTRANS2), Universidad Autónoma de Madrid, Madrid, Spain; 11 Servei de Salut Infantil I de la Dona. Centre Superior d’Investigació en Salut Pública (CSISP). Direcció General de Salut Pública. Conselleria de Sanitat. Generalitat Valenciana, Avda. Catalunya, 21, ES-46020 Valencia, Spain; 12 Universitat Pompeu Fabra, Edifici Mercè Rodoreda - 24, (Campus Ciutadella), C / Ramón Trías Fargas 25-27, ES-08005 Barcelona, Spain

**Keywords:** Employment stability, Unemployment, Temporary contract, Mental health, Gender roles, Marital status, Spain

## Abstract

**Background:**

The growing demand for labour flexibility has resulted in decreasing employment stability that could be associated with poor mental health status. Few studies have analysed the whole of the work force in considering this association since research on flexible forms of employment traditionally analyses employed and unemployed people separately. The gender division of work, and family characteristics related to employment situation, could modify its association with mental wellbeing. The objective of the study was to examine the relationship between a continuum of employment stability and mental health taking into account gender and partner/marital status.

**Methods:**

We selected 6859 men and 5106 women currently salaried or unemployed from the 2006 Spanish National Health Survey. Employment stability was measured through a continuum from the highest stability among employed to lowest probability of finding a stable job among the long-term unemployed. Mental health was measured with the 12-item version of the General Health Questionnaire. Logistic regression models were fitted for each combination of partner/marital status and gender.

**Results:**

In all groups except among married women employment stability was related to poor mental health and a gradient between a continuum of employment stability and mental health status was found. For example, compared with permanent civil servants, married men with temporary contract showed an aOR = 1.58 (95%CI = 1.06–2.35), those working without a contract aOR = 2.15 (95%CI = 1.01–4.57) and aOR = 3.73 (95%CI = 2.43–5.74) and aOR = 5.35 (95%CI = 2.71–10.56) among unemployed of up to two years and more than two years, respectively. Among married and cohabiting people, the associations were stronger among men. Poor mental health status was related to poor employment stability among cohabiting women but not among married ones. The strongest association was observed among separated or divorced people.

**Conclusions:**

There is a rise in poor mental health as the distance from stable employment grows. This result differs according to the interaction with gender and partner/marital status. In Spain this relationship seems to follow a pattern related to the gender division of work in married people but not in other partner/marital situations. Family and socioeconomic context can contribute to explain previous mixed results. Recommendations for research and for action are given.

**Electronic supplementary material:**

The online version of this article (10.1186/s12889-018-5282-3) contains supplementary material, which is available to authorized users.

## Background

Maintaining a stable working life seems to be considered desirable by most workers [1]. Paid work provides the economic resources for individual and family subsistence and also other nonmaterial goods that are a source of wellbeing and health [2]. The deep transformations of industrialized capitalist economies since the 1970s have been characterized by a constant demand for strategies of “labour flexibility” that has meant that jobs are becoming less stable and that long-term employment relationships are becoming less frequent [3].

Research on employment stability and health has focused on two strategies. On one hand there is a well-established body of evidence showing that unemployment is associated with worse mental health and well-being [4, 5]. On the other hand, the threat to job loss among employed people has received increasing interest from diverse scientific fields although no unequivocal results have been obtained [6, 7]. More recently, more complex approaches that analyse the impact on health and wellbeing of flexible employment contexts, consider employment stability as just one dimension of broader constructs [1, 8, 9].

In any case, research on flexible forms of employment is mostly based on approaches which traditionally analyze employed and unemployed people separately. Therefore, a key issue on the research agenda concerns the importance of incorporating integrated perspectives that account for the complexity of different levels of employment stability. One interesting approach in this line is considering employment situation as a continuum that covers the entire work force and ranges from stable work situations to less favourable conditions represented by the loss of employment, passing through a line of worsening conditions [10]. Although some studies have considered this approach [11–13] only few had been conducted in Southern Europe [14, 15].

The characteristics of the context (political, socioeconomic, familiar, cultural) determine the relationship between employment stability and health and wellbeing, as different studies have pointed out. A recent review concluded that conducting studies in different contexts is important for understanding the relationships between unemployment and health outcomes. The authors noted that there are a large number of studies analysing unemployment and health focusing on the effect size at the population level. In contrast to that approach, the review authors recommended a need to conduct analysis of subgroups of populations representing different context, including by gender and for different family and socioeconomic subgroups [16].

Gender differences in employment stability have shown mixed results. Most studies report a greater effect of unemployment on mental health among men [4], although a similar result of both sexes [17] and also a greater association among women was described [18]. A recent study showing worse effects of unemployment on mental health in male from Ireland and similar impact on males and females in Sweden, pointed out the major influence of the context, both family and socioeconomic included, on the relationship between unemployment, gender and mental health [19]. On the other hand, despite women are disproportionately affected by different forms of flexible employment arrangements, the current knowledge about the role of gender in the association of those forms of poor stability and mental health is rather limited [20].

Few studies have taken into account family characteristics in analysing the relationship between employment stability and mental health considering the potentially different gender patterns. A couple of early studies conducted in Catalonia, a Southern European region with a traditional family model (men as breadwinners and women responsible for domestic and family work), suggested that the gender division of the breadwinner and carer roles in the family could modify the association between different levels of employment stability and mental health depending on family arrangements [21, 22]. In countries with traditional family models most unemployed married women have their basic economic needs guaranteed by their husbands’ income. Moreover, women could substitute rewards formerly provided by their job by rewards from the family-nurturing role they play. Additionally, in these countries the association between unemployment and mental health status is much stronger among men due to pressures related to their breadwinner role [22].

In recent decades, research on the relationship between health and marital status, an indicator of family context, has shown that in both sexes married people have better health than single, separated, divorced or widowed people [23]. In addition to economic support, the positive characteristics of social support have been linked to better health among married people [24]. However, recent research has questioned this general acceptance showing different patterns depending on different country’s policies and cultural characteristics regarding family and gender roles [25, 26]. Moreover, studies about partner/marital status and health have been criticized for not taking into account the potential interaction with employment status [26]. Additionally, as far as we know, there are no studies considering the role of partner/marital status in the impact of employment stability on mental health.

Taking into account, then, that the context matters when considering the impact of employment stability on health and wellbeing, analyse the interaction between labour market characteristics and family characteristics as a part of the context can improve the understanding of such relationship. Southern European countries are characterized by a strong labour market regulation, a male breadwinner family model, a low level of support for female participation in the labour force, primary welfare responsibilities lie with the family and policies that help reconcile motherhood and careers are relatively undeveloped [27]. Despite sharing this general socioeconomic context, Spain has a specific market labour characteristic: a high and persistent unemployment rate in combination with precarious employment conditions, including the increasing of temporariness, low salaries, vulnerability and incapacity to exercise rights [28]. Additionally, although labour market participation among Spanish women has increasing in last decades, they have one of the lowest employment rates in Europe as well high unemployment and temporary employment [29]. On the other hand some authors had noted that, in contrast to other Southern European countries, during the last two decades Spain is moving towards a dual-earner family model [30].

The objectives of this study were: 1) to examine the relationship between a continuum of level of employment stability and mental health and 2) to examine whether this association may depend on the interaction with gender and partner/marital status. The level of employment stability refers to the distance from stable employment of the different groups considered and is related to the probability of future stable employment.

## Methods

### Data

The data were taken from the 2006 Spanish National Health Survey, a cross-sectional survey based on a representative sample of the non-institutionalized population of Spain, conducted by the Ministry of Health and Consumer Affairs [31]. The methodology of the survey has been reported elsewhere [25]. For the purposes of this study a subsample of all salaried employees or unemployed people (salaried employees in the last employment), aged 25–64 was selected. To minimize possible reverse causation effects, we excluded those individuals who left work for health reasons (*N* = 120) and those who had never been employed (*N* = 58). The final sample under analysis was composed of 6859 men and 5106 women.

### Measures

*Employment stability* was measured through a combination of three original variables (employment status, type of employment among the employed and time in unemployment among the unemployed). The final variable had the following six categories, ordered from highest employment stability to greatest distance to stable employment: permanent civil servant (“funcionario” in Spanish), permanent contract, temporary contract, working without a contract, unemployed for 2 years or less, unemployed for more than 2 years. The highest category of employment stability, permanent civil servant, is achieved in the public sector after succeeding in the relevant examinations and virtually guarantees employment for the whole working life.

*Mental health status* was measured with the 12-item version of the General Health Questionnaire. This is a screening instrument widely used to detect current, diagnosable psychiatric disorders. It focuses on breaks in normal functioning rather than on lifelong traits; therefore it covers personality disorders or patterns of adjustment when these are associated with distress. The reliability of the test was excellent (Cronbach’s alpha = 0.99). We used a two-point scoring method, rating a problem as absent (0) or present (1) according to the method recommended by the GHQ. The responses were summed, and the participants scoring 3 or more were classified as cases [32].

*Partner/marital status* was based on legal marital status, partner status and living arrangements. It was coded into five categories: married, cohabiting, separated or divorced, single and widowed*.*

### Statistical analysis

First, gender differences for all variables were tested at the bivariate level using the chi-square test for categorical variables and the t-test for age. Second, multiple logistic regression models were fitted in two steps in order to calculate adjusted odds ratios (aOR) and 95% confidence intervals (CI). In the first step models were separated by sex and in the second step by sex and partner/marital status. Models were adjusted for age and occupational social class and weighted by using a calibrated individual weight for the main sample. Occupational social class refers to the respondent’s current occupation, or their last occupation in the case of the unemployed. It was assigned according to a widely used Spanish adaptation of the British classification [33]. The six original social classes were collapsed into two broad categories: I, II and III (non-manual workers), and IVA-IVB-V (manual workers). In addition, to test for an independent linear trend between mental health and employment stability, logistic regression was performed fitting multivariate models including the predictor variable as continuous and using the Wald test.

## Results

### General description of the population

Table 1 shows a general description of the population under study. Employment stability was lower among women. The proportion of women with a permanent contract was lower than that among men, they worked without a contract more frequently, and were more frequently unemployed in any duration considered. The most frequent partner/marital status among both sexes was married, more women than men were separated or divorced while being single was more frequent among men. The proportion of women with poor mental health was greater than that among men.

**Table 1:** General description of the population (in percentages) by sex. Spanish National Health Survey, 2006

	Men *N* = 6859		Women *N* = 5106		*p*
	%		%		
Poor mental health status	13.2		22.7		< 0.001
Employment stability					< 0.001
• Permanent civil servant	9.7		9.5		
• Permanent contract	58.8		45.4		
• Temporary contract	20.4		21.8		
• No contract	1.4		6.6		
• Unemployment = < 2 years	8.3		12.1		
• Unemployment > 2 years	1.4		4.6		
Partner/marital status					< 0.001
• Married	60.8		59.8		
• Cohabiting	10.2		11.9		
• Separated or divorced	2.8		6.8		
• Single	25.7		19.9		
• Widowed	0.5		1.6		
Social class					NS
• Manual	58.3		57.4		
Mean age (SD)	40.21	(10.03)	39.35	(9.55)	< 0.001

### Employment stability and mental health

In both sexes an association between employment stability and mental health was observed and a consistent gradient was found through a continuum from the most stable situation to the greatest distance from stable employment. The magnitude of the association and the gradient were stronger among men. Thus, compared with permanent civil servants, men working without a contract had an aOR = 2.10 (95%CI = 1.13–3.90) while the aOR related to unemployment of up to 2 years and more than 2 years were 3.76 (95%CI = 2.68–5.29) and 6.76 (95%CI = 4.08–11.19), respectively. Among women, the association was similar for working without a contract (aOR = 1.85; 95%CI = 1.28–2.67) and any duration of unemployment (aOR = 1.98; 95%CI = 1.44–2.74 for two or less years of duration and aOR = 1.79; 95%CI = 1.20–2.68 for more than 2 years) (Table 2). Adjusted results showed a small reduction in the association only among women in relation to unadjusted models (Additional file 1: Table S1).

**Table 2:** Association between mental health status and employment stability by sex. Spanish National Health Survey, 2006

	Men *N* = 6972			Women *N* = 5307		
	%	aOR	95% CI	%	aOR	95% CI
Employment stability						
• Permanent civil servant	9.7	1^a^		16.2	1^a^	
• Permanent contract	10.3	1.09	0.81–1.46	20.5	1.23	0.93–1.63
• Temporary contract	14.6	1.63	1.17–2.26**	22.2	1.36	1.00–1.85*
• No contract	17.8	2.10	1.13–3.90*	30.3	1.85	1.28–2.67**
• Unemployment = < 2 years	28.5	3.76	2.68–5.29***	30.7	1.98	1.44–2.74***
• Unemployment > 2 years	41.7	6.76	4.08–11.19***	28.2	1.79	1.20–2.68**

*aOR* Adjusted odds ratio (age and social class). 95% CI = 95% confidence interval

* *p* < 0.05; ** *p* < 0.01; ***p < 0.001

Wald test: ^a^ *p* < 0.05; ^b^ *p* < 0.01; ^c^ *p* < 0.001

### Employment stability and mental health by partner/marital status

Table 3 summarizes the association between employment stability and mental health status, considering the interaction between gender and partner/marital status.

**Table 3:** Association between mental health status and employment stability by partner/marital status. Spanish National Health Survey, 2006

Men															
	Married *N* = 4172			Cohabiting *N* = 699			Separated or divorced *N* = 190			Single *N* = 1766			Widowed *N* = 31		
	%	aOR	95% CI	%	aOR	95% CI	%	aOR	95% CI	%	aOR	95% CI	%	aOR	95% CI
Permanent civil servant	10.7	1^c^		4.2	1^b^		10.5	1^c^		5.9	1^c^		33.3	1	
Permanent contract	10.2	1.00	0.72–1.40	12.0	2.54	0.57–11.42	28.4	3.33	0.57–19.41	8.3	1.31	0.52–3.28	9.5	0.17	0.00–7.33
Temporary contract	15.1	1.58	1.06–2.35*	15.4	3.28	0.70–15.47	9.1	0.65	0.07–5.80	14.1	2.39	0.95–6.06	0.0	0.31	0.00–5014.54
No contract	19.0	2.15	1.01–4.57*	0.0	0.00	0.00-	0.0	0.00	0.00-	22.7	4.40	1.18–16.42*	0.0	0.00	0.00-
Unemployment = < 2 years	28.3	3.73	2.43–5.74***	25.7	7.07	1.37–36.39*	50.0	8.11	1.16–56.48*	27.0	5.34	2.10–13.60***	0.0	0.07	0.00–19.27
Unemployment > 2 years	34.0	5.35	2.71–10.56***	33.3	9.32	0.90–96.33	75.0	51.46	4.50–588.65*	48.5	11.23	3.53–35.75***	0.0	0.00	0.00-
Women															
	Married *N* = 3055			Cohabiting *N* = 608			Separated or divorced *N* = 348			Single *N* = 1014			Widowed *N* = 82		
	%	aOR	95% CI	%	aOR	95% CI	%	aOR	95% CI	%	aOR	95% CI	%	aOR	95% CI
Permanent civil servant	18.9	1		19.4	1^b^		9.4	1^c^		3.9	1^b^		33.3	1	
Permanent contract	20.8	0.93	0.67–1.29	21.2	1.32	0.51–3.41	24.2	2.34	0.65–8.37	15.2	4.33	1.40–13.46*	51.5	2.50	0.47–13.29
Temporary contract	20.5	0.85	0.58–1.25	32.5	2.57	0.95–6.93	41.2	4.68	1.23–17.84*	15.3	4.74	1.47–15.22**	25.0	0.63	0.08–5.14
No contract	28.6	1.20	0.76–1.89	37.0	3.37	1.10–10.33*	33.3	3.38	0.69–16.55	32.5	12.00	3.20–44.96***	11.1	0.46	0.04–5.58
Unemployment = < 2 years	28.6	1.26	0.85–1.88	40.6	3.70	1.29–10.62*	69.7	15.43	3.61–65.89***	19.5	6.15	1.86–20.32**	42.9	1.37	0.18–10.45
Unemployment > 2 years	25.2	1.07	0.64–1.77	33.3	2.20	0.52–9.28	59.1	9.58	2.16–42.47**	22.5	8.57	2.20–33.36**	0.0	0.60	0.02–19.40

*aOR* Adjusted odds ratio (age and social class). 95% CI = 95% confidence interval

* *p* < 0.05; ** *p* < 0.01; ***p < 0.001

Wald test: ^a^ *p* < 0.05; ^b^ *p* < 0.01; ^c^ *p* < 0.001

Among married people employment stability was associated with mental health only among men, with aOR = 1.58 (95%CI = 1.06–2.35) for temporary contracted, aOR = 2.15 (95%CI =1.01–4.57) in case of working without a contract and aOR = 3.73 (95%CI = 2.43–5.74) and aOR = 5.35 (95%CI = 2.71–10.56) for unemployed of up to 2 years and more than 2 years, respectively. Among cohabiting men unemployment was also associated with mental health, with magnitudes greater than for married men (although the difference was not statistically significant in longer-term unemployment). On the other hand, among women in the same group, an association was observed with working without a contract (aOR = 3.37; 95%CI = 1.10–10.33) and, as happened among men although with a lower magnitude, in relation to unemployment (aOR = 3.70; 95%CI = 1.29–10.62 among unemployed for two or less years). In both sexes unemployment was associated with poor mental health among separated or divorced people; this group stands out as having one of the strongest associations between mental health and employment stability. Among single people poor employment stability was positively associated with poor mental health among both sexes; among women all associations were statistically significant, also in the case of permanent contracts, and they presented stronger associations than men in all three groups of employed (for example the aOR among women working without a contract was 12.00; 95%CI = 3.20–44.96 and among men aOR = 4.40; 95%CI = 1.18–16.42).

A gradient was found in all partner/marital status groups except among married women. To facilitate the observation of the gradient these results are graphically shown in Additional file 2: Figure S1. Finally, no association was found among widowed people. Unadjusted models are shown in Additional file 3: Table S2. Adjustment by age and social class changed the magnitude of the associations depending on the group analysed, decreasing in most cases. The most important change was observed in married women among whom working without a contract or being unemployed for two or less years was associated to poor mental health.

## Discussion

The results of this study based on a large and representative sample from Spain are unique in showing the role of partner/marital status in the relationship between a continuum of employment stability and mental health taking into account potential gender differences. It adds to the growing body of evidence indicating that the lack of stable employment can substantially increase the risk of poor mental health and highlights the family and socioeconomic context as potential explanations for previous inconsistent findings. The study deepens in the measurement of employment stability by defining a continuum from the highest stability among employed (as far as we know this is the first study using permanent civil servants as the reference category, an extremely stable situation in Spain) to lowest probability of finding a stable job among the unemployed. Studies about temporary job or unemployment and health usually take stable employment as the reference category, mixing permanent staff with contracted workers, when the stability of the latter group has been declining as more flexible employment policies have been introduced. Our study relied on a nationally representative and large-scale sample making it possible to analyse employment stability across a number of different categories, while also deepening our understanding of the relationship between stability and mental health through stratified models by gender and partner/marital status.

This study has produced four main findings: 1) in all groups, except among married women, the lack of employment stability was related to poor mental health, and a gradient between increasing distance from stable employment and worsening mental health status was found; 2) among married and cohabiting people, the associations were stronger among men; 3) poor mental health status was related to a lack of employment stability among cohabiting women but not among married ones; and 4) the strongest association between lack of employment stability and poor mental health was found in both sexes among separated or divorced people.

### Employment stability and mental health

#### Unemployed people

Unemployment was related to poor mental health status, and a gradient was found related to growing distance to stable employment. These results add to those from the already considerable number of studies analysing unemployment and mental health and are also consistent with studies that have found a positive relationship between length of unemployment and poor mental health status [4]. Regarding employment stability, being unemployed indicates the lowest probability of future stable employment.

Some political and socioeconomic characteristics of the Spanish context could be related to our results, for example, income inequality and the level of unemployment protection. Paul & Moser meta-analysis [4] showed that in countries with high levels of income inequality the negative effects of unemployment on mental health were more severe than in more egalitarian countries. Spain is one of the most unequal European countries (it occupies between the 4th and 6th place in the last 10 years measured by GINI coefficient [14]). Moreover, as other Southern European countries, the unemployment protection is less generous than in other welfare state regimes such as Scandinavian, Bismarckian or Anglo-Saxon. Generosity of unemployment provision has been associated with a positive effect on the mental wellbeing of unemployed people [13, 15].

One of the measures that have been proposed to increase employment is to reduce the rigidity of the Spanish labour market. This measure that could increase the probability of finding a job among unemployed people and perhaps improve the stability of those working without a contract might increase instability among the occupied. In fact, labour reforms carried out in recent years in Spain have introduced greater facilities for dismissal [34]. Although the unemployment rate has decreased, there has been a very sharp deterioration in the working conditions of the employed, and once again temporality has increased [35].

In addition to generous unemployment benefits, active labour market programs (ALMP) could increase the probability of finding a job among the unemployed. Those programs aimed at improving the employability of the unemployed, enhance their possibilities to return to employment. There is some evidence that ALMP have a positive impact on mental health of unemployed people [36], and recent studies suggest that government spending on ALMP may counter the effect of recession on suicide rates [37]. However, it would be better if these programs were also complemented by measures aimed at improving the stability of jobs.

#### Employed people

Among employed people mental health status worsened as stability decreased. Two reviews about temporary employment and psychological health have produced contradictory results. While our results are in accordance with those of Virtanen et al. [7], the review by De Cuyper et al. [38] concluded that research results are inconsistent and inconclusive. Heterogeneity between various temporary arrangements and between countries regarding levels of social protection and workers’ rights may explain some of the mixed findings [8]. Carr demonstrated that Mediterranean countries (including Spain), where there is not much support for the unemployed either through benefits or activation measures, insecure employment has a much stronger negative impact on life satisfaction [6]. A qualitative study conducted in Spain suggested that probably the main disadvantage of temporary work is the uncertainty about maintaining employment and the difficulties finding a new one in a country with very high unemployment rates [39].

The worst mental health among employees was found in people working without a contract. A study conducted in Catalonia reported that among manual workers, both men and women, working with no contract was related to poor mental health [21]. In addition, this form of informal employment is associated with other harmful conditions of vulnerability such as exploitation, arbitrary relationships and denial of basic rights of workers [40].

Using a variable that measures from the maximum stability at work to the lowest probability of future stable employment among the unemployed, suggests that an important part of the association is explained by the uncertainty of recovering or maintaining an employment. However, it is necessary to consider that lack of stability is just one dimension of broader constructs such as precarious employment and therefore the poorest situation of employees in different situations of low stability could be explained not only by the fear of losing their jobs but also by the poorer conditions related with precarious employment [8, 41].

### Employment stability, mental health and partner/marital status

#### Married people

Among married men decreasing employment stability increased the likelihood of their having poor mental health. In contrast, married women were the only group showing no association between employment stability and mental health. Our study was conducted in Spain, a country characterized by a traditional family model. These results go in line with those found in Ireland [19], a country with a similar family model, suggesting a pattern in relation to the deleterious effect on mental health of the role of breadwinner in case of employment instability. The pressure to continue providing the financial resources at home among working men, and the uncertainty, among the unemployed, of not knowing when they will regain that role would explain the results among married men. Additionally, the breadwinner role among employed married men in more unstable jobs could lead them to accept more vulnerable situations in order to keep the job. It has been reported that married men work longer hours, work harder, have lower absenteeism and were more obedient to their superiors than single men [42]. Artazcoz et al. proposed a model to explain the deleterious effect on health of the acceptance of working long hours and other poor working conditions among breadwinners [43].

Married women, in contrast, have access to alternative roles (for example child rearing) that may be able to serve as substitutes for employment, at least to a certain degree. Moreover, unemployed married women can expect more financial support from their husbands than unemployed men from their wives, as men still earn more than women.

#### Cohabiting people

Although many studies about marital status and health have considered married and cohabiting partners as one category, we found poorer health outcomes among cohabiting people, both men and women. Even though the difference was stronger among women since the association of employment stability found among cohabiting was not found in married ones. The weaker commitment and lower stability characteristic of cohabiting unions [44] may heighten instability, adding to that related to their employment situation. So the threat of job loss or the difficulty to find a new job among unemployed people with a weak partner relationship and the accompanying fear of losing a source of financial and social support could explain the poorer situation among them. It should be noted that cohabitation has gone from a marginal role in the family formation process to a significantly spreading into the Spanish population. In fact, in our study this situation accounted for around 10% of people.

#### Separated or divorced

The higher prevalence of poor mental health was observed in separated or divorced unemployed people of both sexes. These results add to the extensive body of literature reporting that separated and divorced people have poorer mental health than their married counterparts [45]. Part of this association among people in the labour force could be related to poor employment stability.

Having children can be one source of family financial stress among separated or divorced people with less probability of future stable employment, especially those unemployed. Some men must pay child support payments for the maintenance of their children and also maintain their own home, since in most cases child custody and family housing is assumed by the former partner. However, it is possible that this situation is not the most frequent, since only 20.4% of men in this group had children (dependent or not). Among separated or divorced women with children in their care, the threat to employment stability could partly explain their poor mental health (in our study 77.7% of separated or divorced women had at least one child). Unemployed and temporarily contracted women showed the strongest association with poor mental health. The economic vulnerability of being a breadwinner in the case of separated or divorced women could oblige them to accept poor working conditions in order to avoid losing their job similar to the mechanism mentioned previously in married men. Additionally, although a selection bias cannot be ruled out, the higher magnitudes of the OR found in this group could be also related to the lower than average wage replacement rates among unemployed women.

#### Single people

In both sexes low employment stability was associated with poorer mental health status among single people following a continuum from stable employment to the greatest distance from that situation. Additionally, employed women showed stronger associations than men that could be related, in addition to fear of losing their job, with poorer working conditions frequently observed among women. Women in situations of lower employment stability (working without a contract) presented the strongest association, probably mixing the relationships between poor stability and other poor working conditions characteristic of precariousness, which are more frequent among women [20]. It should be noted that domestic workers and cleaners accounted for 70.0% of this group, among whom it is well known that working and employment conditions are poor.

In all groups of men, long-term unemployed showed worse mental health than short-term unemployed. Among women, while singles showed a similar association, cohabiting and being separated or divorced presented a reverse relationship. These two patterns of association are described in the literature: a) an increase in poor mental health as time in unemployment increases [4, 46], and b) a levelling off or even slight recovery in wellbeing, interpreted as a result of the unemployed beginning to face and adapt to their challenging circumstances [4]. The different gender pattern that we find suggests that the alternative role of caring performed by cohabiting and separated or divorced women (the majority of whom had at least one child) could contribute to a certain adaptation to long term unemployment, while single women would not have this role.

#### Widowed

No associations were found between employment stability and mental health probably because lack of statistical power due to the few people in this situation.

### Limitations

The possibility of reverse causation must be considered. We have tried to reduce this possibility among unemployed people by excluding people who were unemployed for health reasons, as well as those never employed. However, probably some effect remains, since often illness or mild-moderate mental health issues are secondary issues in job loss. It must be taken into account, however, that the meta-analysis conducted by Paul & Moser concluded that the practical importance of this effect might be limited [4]. One the other hand, reverse causation can explain, at least in part, the association between mental health and other levels of employment stability, given the capacity of mental health to predict employment opportunities and stability. Another limitation is that we have not taken into account the possible interaction of social class because few people in low social positions worked as permanent civil servants. Since we adjusted for this variable, we could not observe inequalities related to social position, but we were able to clarify the interaction between gender and partner/marital status.

A high unemployment rate, fast deterioration of the stability and quality of jobs and cuts in social and employment policies as a result of the last economic crisis in Spain, could change the results found in the present study, likely worsening the situation of people in unstable employment situations [47]. Some studies analysing the effect of the economic crisis in mental health in Spain suggest that unemployment was related to an increase in poor mental health among men [48, 49], mainly among breadwinners [50]. In the case of women, one study suggested that the poorest mental health among women from Southern European countries was related to changes in their employment status. Due to the increase of unemployment, primarily among men during the first phase of the crisis, many partnered women were pushed to enter the labour market forced by their economic needs and vulnerability that reduced their bargaining power for better employment conditions, including stability [51]. Moreover, Urbanos et al. demonstrated worsening mental health among unemployed Spaniards during the economic crisis, mainly among the long term unemployed; unfortunately, the study didn’t take into account gender differences [52].

## Conclusions

The relationship between employment stability and mental health follows a continuum from more stable situations to lowest probability of future stable employment associated to from better to poorer mental health status. This result differs according to the interaction with gender and partner/marital status. In Spain this relationship seems to follow a pattern related to the gender division of work in married people but not in other partner/marital situations. Family and socioeconomic context can contribute to explain the results.

The recommendations for future research include a) design longitudinal studies in order to take into account changing labour market trajectories and people’s increasingly complex partner/marital life course, and b) consider how the relation between employment stability and mental health, considering gender and partner/marital status, may vary across socio-economic groups. The recommendations for action are: a) implement generous unemployment benefit programs and invest in active labour market programs, giving the capacity to improve mental wellbeing among not only the unemployed [36, 53, 54], but also among employed people [55], b) stimulate stability of employment relations, c) define policies that promote and protect social ties within the Spanish context to offset some of the health impact of employment stability; some of the benefits might be specific to family situations [24], and d) take gender and family status into consideration when designing social policies to address economic and labour market changes.

## Additional files

Additional file 1: **Table S1.** Unadjusted association between mental health status and employment stability by sex. Spanish National Health Survey, 2006. (DOCX 19 kb)

Additional file 2: **Figure S1.** Association between mental health status and employment stability by partner/marital status among men and women. Spanish National Health Survey, 2006. (DOCX 112 kb)

Additional file 3: **Table S2.** Unadjusted association between mental health status and employment stability by partner/marital status. Spanish National Health Survey, 2006. (DOCX 26 kb)

## Abbreviations

aOR: Adjusted odds ratio
CI: Confidence interval

## References

[1] Davoine L, Erhel C, Guergoat-Lariviere M. Monitoring quality in work: European Employment Strategy indicators and beyond. Int. Labour Rev. [Internet]. 2008;147:163–198. Available from: file:///C:/Users/HP/Downloads/0c9605247f7ba094c3000000.pdf.

[2] Jahoda M (1981). Work, employment, and unemployment: values, theories, and approaches in social research. Am Psychol.

[3] Boyer R, Buechtemann CF (1993). The economics of job protection and emerging new capital labour relations. Employ. Secur. Labour mark. Behav. Interdiscip. Approaches Int. Evid.

[4] Paul KI, Moser K. Unemployment impairs mental health: Meta-analyses. J. Vocat. Behav. [Internet]. Elsevier Inc.; 2009;74:264–282.[cited 24 July 2014] Available from: https://www.sciencedirect.com/science/article/abs/pii/S0001879109000037.

[5] Rueda S, Chambers L, Wilson M, Mustard C, Rourke SB, Bayoumi A, et al. Association of returning to work with better health in working-aged adults: a systematic review. Am J Public Health. 2012;102:541–56. 10.2105/AJPH.2011.300401 PMC3487667 22390520

[6] Carr E, Chung H. Employment insecurity and life satisfaction: The moderating influence of labour market policies across Europe. J Eur Soc Policy. 2014;24:383–99.

[7] Virtanen M, Kivimäki M, Joensuu M, Virtanen P, Elovainio M, Vahtera J. Temporary employment and health: a review. Int J Epidemiol. 2005;34:610–22. 10.1093/ije/dyi024 15737968

[8] Benach J, Vives a, Amable M, Vanroelen C, Tarafa G, Muntaner C. Precarious employment: understanding an emerging social determinant of health. Annu Rev Public Health. 2014;35:229–53. 10.1146/annurev-publhealth-032013-182500 24641559

[9] Burchell B, Sehnbruch K, Piasna A, Agloni N. The quality of employment and decent work: definitions, methodologies, and ongoing debates. Cambridge J Econ. 2013;38:459–77.

[10] Dooley D (2003). Unemployment, underemployment, and mental health: conceptualizing employment status as a continuum. Am J Community Psychol.

[11] Grzywacz JG, Dooley D. ‘Good jobs’ to ‘bad jobs’: replicated evidence of an employment continuum from two large surveys. Soc Sci Med. 2003;56:1749–60. https://s3.amazonaws.com/academia.edu.documents/45537027/37.pdf?AWSAccessKeyId=AKIAIWOWYYGZ2Y53UL3A&Expires=1521323031&Signature=oL7daqRjmtPTKF9bkxO7RdB5qZw%3D&responsecontent-disposition=inline%3B%20filename%3DGood_jobs_to_bad_jobs_replicated_eviden.pdf. 10.1016/s0277-9536(02)00170-3 12639591

[12] Leach LS, Butterworth P, Strazdins L, Rodgers B, Broom DH, Olesen SC. The limitations of employment as a tool for social inclusion. BMC Public Health. BioMed Central Ltd. 2010;10:621. 10.1186/1471-2458-10-621 PMC2972242 20955623

[13] Virtanen P, Liukkonen V, Vahtera J, Kivimaki M, Koskenvuo M. Health inequalities in the workforce: the labour market core-periphery structure. Int J Epidemiol. 2003;32:1015–21. https://academic.oup.com/ije/article/32/6/1015/775166. 10.1093/ije/dyg319 14681267

[14] Sánchez-moreno E, Gallardo-peralta LP, Barrón A, Arias-astray A (2016). Employment status and health in Spain before and after the great recession. Soc Curr.

[15] Fiori F, Rinesi F, Spizzichino D, Di Giorgio G. Employment insecurity and mental health during the economic recession: An analysis of the young adult labour force in Italy. Soc Sci Med. 2016;153:90–8. 10.1016/j.socscimed.2016.02.010 26889951

[16] Norström F, Virtanen P, Hammarström A, Gustafsson PE, Janlert U (2014). How does unemployment affect self-assessed health ? A systematic review focusing on subgroup effects. BMC Public Health.

[17] Thomas C, Benzeval M, Stansfeld S a. Employment transitions and mental health: an analysis from the British household panel survey. J Epidemiol. Community Health. 2005;59:243–9. 10.1136/jech.2004.019778 PMC1733037 15709086

[18] Frasquilho D, de Matos M, Marques A, Gaspar T, Caldas-de-Almeida J. Distress and unemployment: the related economic and noneconomic factors in a sample of unemployed adults. Int J Public Health. 2016:1–8. 10.1007/s00038-016-0806-z 26971795

[19] Strandh M, Hammarström A, Nilsson K, Nordenmark M, Russel H (2013). Unemployment, gender and mental health : the role of the gender regime. Sociol Health Illn.

[20] Menéndez M, Benach J, Muntaner C, Amable M, O’Campo P. Is precarious employment more damaging to women’s health than men’s? Soc. Sci Med. 2007;64:776–81. 10.1016/j.socscimed.2006.10.035 17140717

[21] Artazcoz L, Benach J, Borrell C, Cortès I. Social inequalities in the impact of flexible employment on different domains of psychosocial health. J Epidemiol Community Health. 2005;59:761–7. 10.1136/jech.2004.028704 PMC1733125 16100314

[22] Artazcoz L, Benach J, Borrell C, Cortès I. Unemployment and Mental Health: Understanding the Interactions Among Gender, Family Roles, and Social Class. Am J Public Health. 2004;94:82–8. 10.2105/ajph.94.1.82 PMC1449831 14713703

[23] Lund R, Due P, Modvig J, Holstein BE, Damsgaard MT, Andersen PK. Cohabitation and marital status as predictors of mortality—an eight year follow-up study. Soc Sci Med. 2002;55:673–9. https://pdfs.semanticscholar.org/7c16/bcd73b0e15165055067366a89a59f2d471f1.pdf. 10.1016/s0277-9536(01)00219-2 12188471

[24] Umberson D, Montez JK (2010). Social relationships and health: a flashpoint for health policy. J Health Soc Behav.

[25] Artazcoz L, Cortès I, Borrell C, Escribà-Agüir V, Cascant L. Social inequalities in the association between partner/marital status and health among workers in Spain. Soc Sci Med. 2011;72:600–7. 10.1016/j.socscimed.2010.11.035 21211876

[26] Simon RW. Revisiting the relationships among gender, marital status, and mental health. Publications Office of the European Union. Am J Sociol. 2002;107:1065–96. 10.1086/339225 12227382

[27] Ferrera M (1996). The “southern model” of welfare in social Europe. J. Eur. Soc Policy.

[28] Vives A, Amable M, Ferrer M, Moncada S, Llorens C, Muntaner C, et al. Employment precariousness and poor mental health: evidence from Spain on a new social determinant of health. J Environ Public Health. 2013;2013:1–10. 10.1155/2013/978656 PMC3574746 23431322

[29] Eurofound. The gender employment gap: challenges and solutions. Luxembourg; 2016.

[30] Naldini M, Jurado T (2013). Family and Welfare state reorientation in Spain and inertia in Italy from a European perspective. Popul Rev.

[31] Ministerio de Sanidad y Consumo (2006). Metodologia de la Encuesta Nacional de Salud de.

[32] Goldberg D (1978). Manual of the general health questionnaire.

[33] Domingo-Salvany A, Regidor E, Alonso J, Álvarez-Dardet C. Una propuesta de medida de la clase social: Grupo de Trabajo de la Sociedad Española de Epidemiología y de la Sociedad Española de Medicina familiar y Comunitaria [proposal for a social class measure: working group of the Spanish society epidemiology and. Atención Primaria 2000;25:350–363. 10905823

[34] Clauwaert S, Schömann I (2012). The crisis and national labour law reforms: a mapping exercise.

[35] Miguélez F, Alós R, Carrasquer P, Lope A, Molina Ó, Pastor A, et al. [Diagnóstico socio-económico sobre las políticas de empleo en España, 2012–2014] [Internet]. Centre d’Estudis Sociològics sobre la Vida Quotidiana i el Treball – QUIT Institut d’Estudis del Treball, editor. Bellaterra: Universitat Autònoma de Barcelona; 2015. Available from: https://ddd.uab.cat/record/142865.

[36] Coutts AP (2009). Active labour market Programmes ( ALMPs ) and health : an evidence-bas. Review prepared for the strategic review of health inequalities in England post 2010 ( marmot review ).

[37] Stuckler D, Basu S, Suhrcke M, Coutts A, Mckee M (2009). The public health eff ect of economic crises and alternative policy responses in Europe : an empirical analysis. Lancet [internet].

[38] De Cuyper N, de Jong J, De Witte H, Isaksson K, Rigotti T, Schalk R. Literature review of theory and research on the psychological impact of temporary employment: Towards a conceptual model. Int J Manag Rev. 2008;10:25–51.

[39] Amable M, Benach J, González S (2001). La precariedad laboral y su repercusión sobre la salud: conceptos y resultados preliminares de un estudio multimétodos. Arch Prev Riesgos Labor.

[40] Porthé V, Benavides FG, Vázquez ML, Ruiz-Frutos C, García AM, Ahonen E, et al. [Precarious employment in undocumented immigrants in Spain and its relationship with health]. Gac Sanit. 2009;23(Suppl 1):107–14. 10.1016/j.gaceta.2009.09.004 19913332

[41] Benach J, Muntaner C. Precarious employment and health: developing a research agenda. J Epidemiol Community Health. 2007;61:276–7. 10.1136/jech.2005.045237 PMC2652930 17372284

[42] Ahituv A, Lerman RI. Job turnover, wage rates, and marital stability: How are they related? Rev Econ Househ. 2010;9:221–49.

[43] Artazcoz L, Cortès I, Escribà-Agüir V, Cascant L, Villegas R. Understanding the relationship of long working hours with health status and health-related behaviours. J Epidemiol Community Health. 2009;63:521–7. 10.1136/jech.2008.082123 19254912

[44] Brown SL, Booth A (1996). Cohabitation versus marriage: a comparison of relationship quality. J Marriage Fam.

[45] Kessler RC, Bromet EJ (2013). The epidemiology of depression across cultures. Annu Rev Public Heal.

[46] Bambra C, Eikemo T A. Welfare state regimes, unemployment and health: a comparative study of the relationship between unemployment and self-reported health in 23 European countries. J Epidemiol Community Health. 2009;63:92–98. 10.1136/jech.2008.077354 18930981

[47] Cortès-Franch I, González López-Valcárcel B. [The economic-financial crisis and health in Spain. Evidence and viewpoints. SESPAS report 2014]. Gac Sanit. 2014;28(Suppl 1):1–6. 10.1016/j.gaceta.2014.03.011 24863987

[48] Lopez-Bernal J, Gasparrini A, Artundo L, McKee M (2013). The effect of the late 2000s financial crisis on suicides in Spain: an interrupted time-series analysis. Eur J Pub Health.

[49] Gili M, Roca M, Basu S, McKee M, Stuckler D (2013). The mental health risks of economic crisis in Spain: evidence from primary care centres, 2006 and 2010. Eur J Pub Health.

[50] Bartoll X, Palència L, Malmusi D, Suhrcke M, Borrell C (2013). The evolution of mental health in Spain during the economic crisis. Eur J Pub Health.

[51] Artazcoz L, Cortès I, Puig-Barrachina V, Benavides FG, Escribà-Agüir V, Borrell C. Combining employment and family in Europe: the role of family policies in health. Eur J Public Health. 2014;24:649–55. 10.1093/eurpub/ckt170 24213585

[52] Urbanos-Garrido RM, Lopez-valcarcel BG (2015). The influence of the economic crisis on the association between unemployment and health: an empirical analysis for Spain. Eur J Health Econ.

[53] Ferrarini T, Sjöberg O (2010). Social policy and health: transition countries in a comparative perspective. Int J Soc Welf.

[54] O’Campo P, Molnar A, Ng E, Renahy E, Mitchell C, Shankardass K (2015). Social welfare matters : a realist review of when, how, and why unemployment insurance impacts poverty and health. Soc Sci Med Elsevier Ltd.

[55] Dragano N, Siegrist J, Wahrendorf M, Dragano N, Siegrist J, Wahrendorf M (2011). Welfare regimes, labour policies and unhealthy psychosocial working conditions : a comparative study with 9917 older employees from 12 European countries. J Epidemiol Community Health.

