# Supplementary material for: Employment stability and mental health in Spain: towards understanding the influence of gender and partner/marital status
Source: BMC Public Health. 2018 Apr 2;18:425. doi: 10.1186/s12889-018-5282-3 (PMC5879603; doi:10.1186/s12889-018-5282-3)
Supplement: Supplementary file 1 — Table S1. Unadjusted association between mental health status and employment stability by sex. Spanish National Health Survey, 2006. (DOCX 19 kb) [file 12889_2018_5282_MOESM1_ESM.docx]

Additional file 1

Table S1. Unadjusted association between mental health status and employment stability by sex. Spanish National Health Survey, 2006.

|  | Men  N=6972 | | | Women  N=5307 | | |
| --- | --- | --- | --- | --- | --- | --- |
|  | % | OR | 95% CI | % | OR | 95% CI |
| Employment stability |  |  |  |  |  |  |
| - Permanent civil servant | 9.7 | 1^a^ |  | 16.2 | 1^a^ |  |
| - Permanent contract | 10.3 | 1.08 | 0.81-1.43 | 20.5 | 1.33 | 1.02-1.73* |
| - Temporary contract | 14.6 | 1.61 | 1.18-2.18** | 22.2 | 1.47 | 1.11-1.96** |
| - No contract | 17.8 | 2.01 | 1.10-3.67* | 30.3 | 2.24 | 1.60-3.15*** |
| - Unemployment =< 2 years | 28.5 | 3.73 | 2.70-5.15*** | 30.7 | 2.28 | 1.69-3.08*** |
| - Unemployment > 2 years | 41.7 | 6.77 | 4.18-10.99*** | 28.2 | 2.05 | 1.40-3.01*** |

OR = odds ratio. 95% CI = 95% confidence interval.

* p <0.05; ** p<0.01; ***p<0.001

Wald test: ^a^ p <0.05; ^b^ p<0.01; ^c^ p<0.001
