# Supplementary material for: Employment stability and mental health in Spain: towards understanding the influence of gender and partner/marital status
Source: BMC Public Health. 2018 Apr 2;18:425. doi: 10.1186/s12889-018-5282-3 (PMC5879603; doi:10.1186/s12889-018-5282-3)
Supplement: Supplementary file 2 — Figure S1. Association between mental health status and employment stability by partner/marital status among men and women. Spanish National Health Survey, 2006. (DOCX 112 kb) [file 12889_2018_5282_MOESM2_ESM.docx]

**Additional file 2**

**Figure S1. Association between mental health status and employment stability by partner/marital status among men and women. Spanish National Health Survey, 2006.**


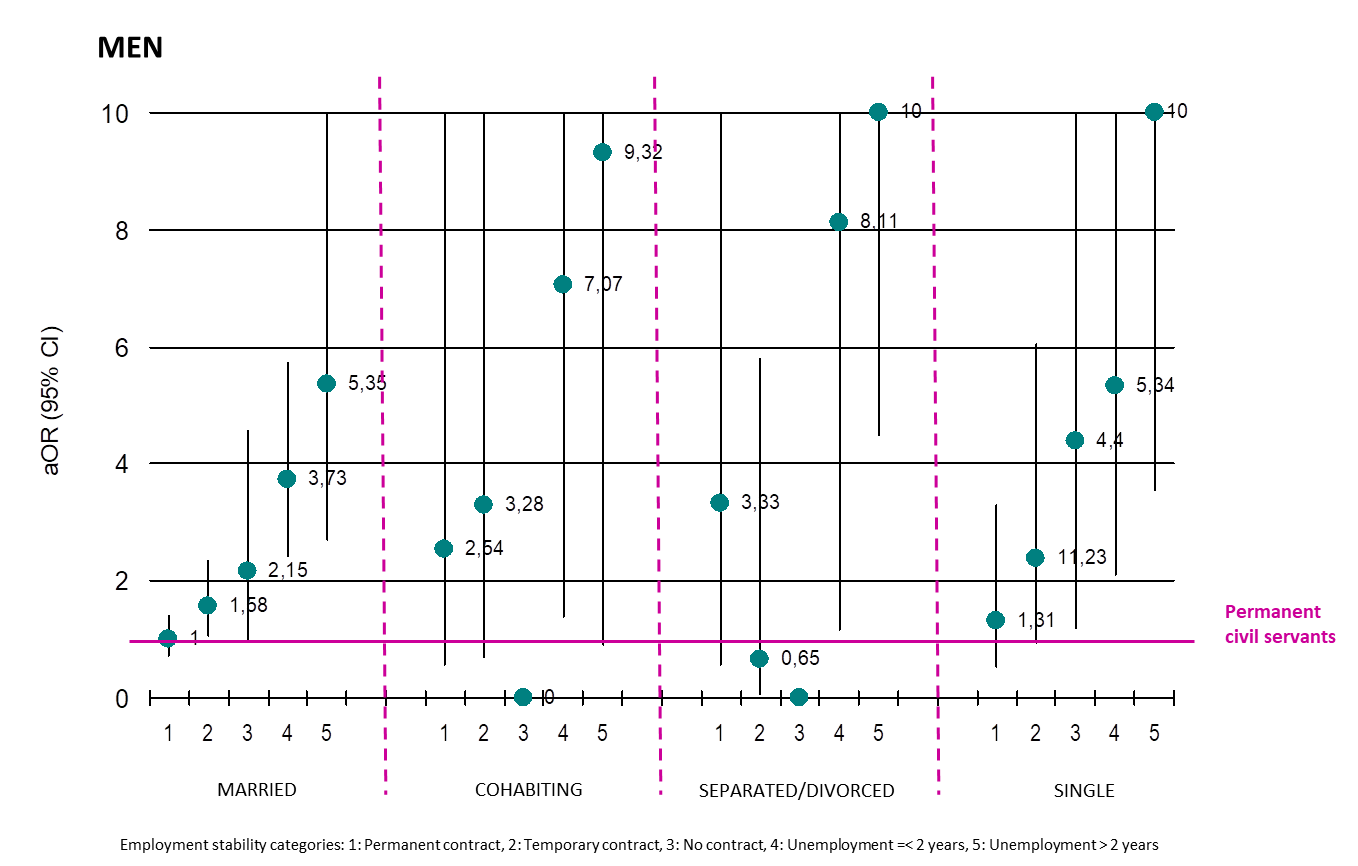


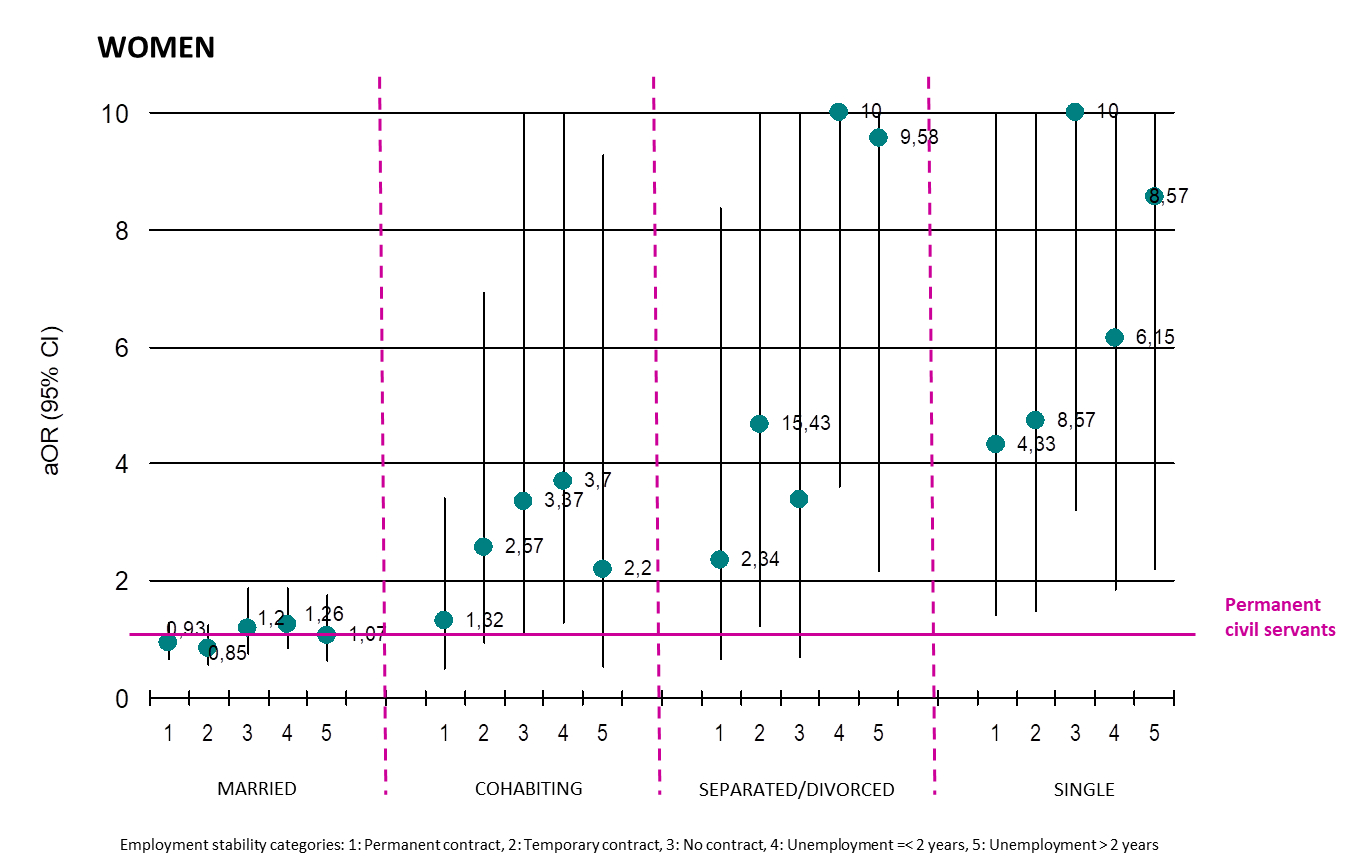


Note: the upper IC limit has been truncated in value 10 for graphic reasons.
