# Supplementary material for: Employment stability and mental health in Spain: towards understanding the influence of gender and partner/marital status
Source: BMC Public Health. 2018 Apr 2;18:425. doi: 10.1186/s12889-018-5282-3 (PMC5879603; doi:10.1186/s12889-018-5282-3)
Supplement: Supplementary file 3 — Table S2. Unadjusted association between mental health status and employment stability by partner/marital status. Spanish National Health Survey, 2006. (DOCX 26 kb) [file 12889_2018_5282_MOESM3_ESM.docx]

**Additional file 3**

**Table S2. Unadjusted association between mental health status and employment stability by partner/marital status. Spanish National Health Survey, 2006.**

| **MEN** |  |  | |  |  | |  |  | |  |  | |  |  | |
| --- | --- | --- | --- | --- | --- | --- | --- | --- | --- | --- | --- | --- | --- | --- | --- |
|  | Married  N=4172 | | | Cohabiting  N=699 | | | Separated or divorced  N=190 | | | Single  N=1766 | | | Widowed  N=31 | | |
|  | % | OR | 95% CI | % | OR | 95% CI | % | OR | 95% CI | % | OR | 95% CI | % | OR | 95% CI |
| Permanent civil servant | 10.7 | 1^c^ |  | 4.2 | 1^b^ |  | 10.5 | 1^c^ |  | 5.9 | 1^c^ |  | 33.3 | 1 |  |
| Permanent contract | 10.2 | 0.95 | 0.69-1.31 | 12.0 | 3.25 | 0.74-14.26 | 28.4 | 4.31 | 0.79-23.71 | 8.3 | 1.35 | 0.55-3.31 | 9.5 | 0.18 | 0.12-7.72 |
| Temporary contract | 15.1 | 1.49 | 1.04-2.15* | 15.4 | 4.41 | 0.98-19.77 | 9.1 | 1.13 | 0.15-8.44 | 14.1 | 2.40 | 0.97-5.92 | 0.0 | 0.39 | 0.00-2498.94 |
| No contract | 19.0 | 1.87 | 0.90-3.87 | 0.0 | 0.00 | 0.00- | 0.0 | 0.00 | 0.00- | 22.7 | 4.51 | 1.23-16.53* | 0.0 | 0.00 | 0.00- |
| Unemployment =< 2 years | 28.3 | 3.31 | 2.21-4.97*** | 25.7 | 8.48 | 1.66-43.23* | 50.0 | 11.29 | 1.86-68.50* | 27.0 | 5.46 | 2.20-13.58*** | 0.0 | 0.07 | 0.00-9.85 |
| Unemployment > 2 years | 34.0 | 4.36 | 2.24-8.48*** | 33.3 | 11.38 | 1.14-114.12* | 75.0 | 38.37 | 3.77-390.42* | 48.5 | 13.96 | 4.62-42.21*** | 0.0 | 0.00 | 0.00- |
| **WOMEN** |  |  | |  |  | |  |  | |  |  | |  |  | |
|  | Married  N=3055 | | | Cohabiting  N=608 | | | Separated or divorced  N=348 | | | Single  N=1014 | | | Widowed  N=82 | | |
|  | % | OR | 95% CI | % | OR | 95% CI | % | OR | 95% CI | % | OR | 95% CI | % | OR | 95% CI |
| Permanent civil servant | 18.9 | 1^c^ |  | 19.4 | 1^c^ |  | 9.4 | 1^c^ |  | 3.9 | 1^b^ |  | 33.3 | 1 |  |
| Permanent contract | 20.8 | 1.13 | 0.83-1.54 | 21.2 | 1.08 | 0.45-2.60 | 24.2 | 3.02 | 0.88-10.40 | 15.2 | 3.80 | 1.24-11.61* | 51.5 | 2.34 | 0.48-11.36 |
| Temporary contract | 20.5 | 1.10 | 0.78-1.56 | 32.5 | 1.95 | 0.81-4.70 | 41.2 | 6.59 | 1.83-23.65** | 15.3 | 3.85 | 1.24-11.99* | 25.0 | 0.61 | 0.08-4.42 |
| No contract | 28.6 | 1.73 | 1.15-2.61** | 37.0 | 2.36 | 0.88-6.35 | 33.3 | 5.05 | 1.10-23.24* | 32.5 | 9.81 | 2.74-35.11*** | 11.1 | 0.37 | 0.04-3.76 |
| Unemployment =< 2 years | 28.6 | 1.73 | 1.20-2.48** | 40.6 | 2.78 | 1.08-7.17* | 69.7 | 22.69 | 5.59-92.19*** | 19.5 | 5.09 | 1.58-16.42** | 42.9 | 1.66 | 0.28-9.95 |
| Unemployment > 2 years | 25.2 | 1.43 | 0.89-2.32 | 33.3 | 1.99 | 0.54-7.21 | 59.1 | 13.17 | 3.09-56.18*** | 22.5 | 5.94 | 1.58-22.26** | 0.0 | 0.54 | 0.02-16.98 |

OR = odds ratio. 95% CI = 95% confidence interval.

* p <0.05; ** p<0.01; ***p<0.001.

Wald test: ^a^ p <0.05; ^b^ p<0.01; ^c^ p<0.001
